# Advanced soilless cultivation of medicinal plants: integrating dynamic LED lighting, elicitation, and smart technologies for sustainable biomass and phytochemical production

**DOI:** 10.3389/fpls.2026.1970412

**Published:** 2026-10-12

**Authors:** Amit Kumar, Kanika, Shashi Rani, Ashish Rambhau Warghat

**Affiliations:** 1 Cell and Tissue Engineering Laboratory, Fermentation and Phytofarming Technology Division, CSIR-Institute of Himalayan Bioresource Technology, Palampur, Himachal Pradesh, India; 2 Academy of Scientific and Innovative Research (AcSIR), Ghaziabad, India

**Keywords:** controlled environment agriculture, medicinal plants, nutrient management, secondary metabolites, vertical farming

## Abstract

Countries with limited water and land resources confront a significant problem due to the growing demand for medicinal plants. These plants are used worldwide to extract and isolate therapeutically important bioactive molecules, known as secondary metabolites. Many medicinal plant species are currently facing significant conservation challenges due to excessive, unregulated harvesting, driven by rapidly growing demand for plant-derived therapies. Soilless farming techniques like hydroponics, aeroponics, and vertical farming offer a sustainable alternative for generating high-quality planting material on a large scale in a considerably shorter period and with less space. Soilless farming revolutionises agriculture and food production, making it particularly well-suited for urban agriculture by reducing maintenance requirements and maximizing crop yields. Soilless farming of numerous medicinal herbs, fruit crops, and vegetables has become increasingly popular in recent years, particularly for its ability to produce large amounts of biomass, high yields, and a wide range of pharmacologically active metabolites. These high-tech systems have significant promise for increased crop yields, cleaner produce, season-independent cultivation, and reduced environmental impact, particularly in water use and disease, compared to traditional soil-based systems. This review article rigorously evaluates recent advancements in soilless agriculture, including LED spectral regulation, nano-elicitation, microbial biostimulants and precursor-feeding strategies, reusable substrates, computational technologies, and CRISPR/Cas-based editing, with respect to their efficacy in generating high-quality plant biomass, improving automation, and optimizing shoot architecture. Implementing soilless farming to produce and synthesize bioactive molecules is expected to be crucial in enhancing sustainability and human well-being.

## Introduction

In recent decades, pharmaceutical companies have paid much attention to plant-derived bioactive molecules for their pharmacological activities and few side effects (Nasim et al., 2022; Direito et al., 2024). A variety of phytochemicals, such as terpenoids, flavonoids, and alkaloids, are produced and accumulated in different parts of medicinal plants, contributing to their therapeutic properties and health benefits (Stéphane et al., 2022; Chaachouay et al., 2023). These secondary metabolites affect the plant’s performance, directly or indirectly, and help it adapt to stressful environments, ensuring its survival in unfavorable conditions. The biosynthesis of secondary metabolites in plants is affected by various factors, *viz*., light, temperature, humidity, the season of cultivation, genetic and genotypic selection, plant age, crop type, and environmental conditions encountered during plant growth and development (Partap et al., 2020). The herbal, pharmaceutical, and food sectors rely entirely on organically grown plants or conventional agricultural processes for large-scale growth. Traditional soil-based agricultural methods fail to meet the intended requirements and to address problems associated with high costs, microbiological contamination, soil particle accumulation, toxic metal presence, and other forms of adulteration in raw plant materials. Efforts are underway to explore alternative cultivation methods to ensure the global food supply while meeting industry requirements for large-scale, high-yield production (Jaeger et al., 2025). For this reason, alternative soilless growing techniques, such as vertical farming and hydroponics, have been developed to enable large-scale production of commercially significant plants under controlled conditions. These high-tech alternative systems have the potential to enhance the quality, purity, consistency, bioactivity, and biomass yield of raw materials and provide a reliable, eco-friendly foundation for cultivating economically valuable crops, such as medicinal plants, floral herbs, green vegetables, and spices. Soilless farming in a controlled environment can produce leaf and root material of superior quality, free from unwanted substances such as weeds, soil, and environmental pollutants like heavy metals (Kumar et al., 2025a; Kanika et al., 2025).

Growing plants without soil and with reduced water usage in vertically stacked layers is known as vertical farming, which increases agricultural productivity per unit area (Jia et al., 2024). Hydroponics is a method of growing plants using mineral nutrient solutions in water without soil. Vertical farming and hydroponics are modern farming methods in which all environmental parameters and biofortification for value addition are precisely regulated and monitored (Erekath et al., 2024). These high-tech techniques enable the production of food and plants in urban areas and offer benefits such as enhanced protection against diseases, pests, and drought, consistent crop output year-round, and reduced transportation costs (Erekath et al., 2024). Rather than draining into the soil, the nutrient-enriched water is collected and recirculated in a “closed loop” system, eliminating water waste and nutrient/fertilizer runoff. Numerous studies have shown that plants cultivated in soilless systems exhibit higher productivity and phytochemical content compared with plants grown in soil (Verdoliva et al., 2021; Fatima et al., 2023). Comparison of soil-based and soilless farming regarding production, nutrients, water use, and resource management efficiency (**Figure 1**).

**Figure 1:**
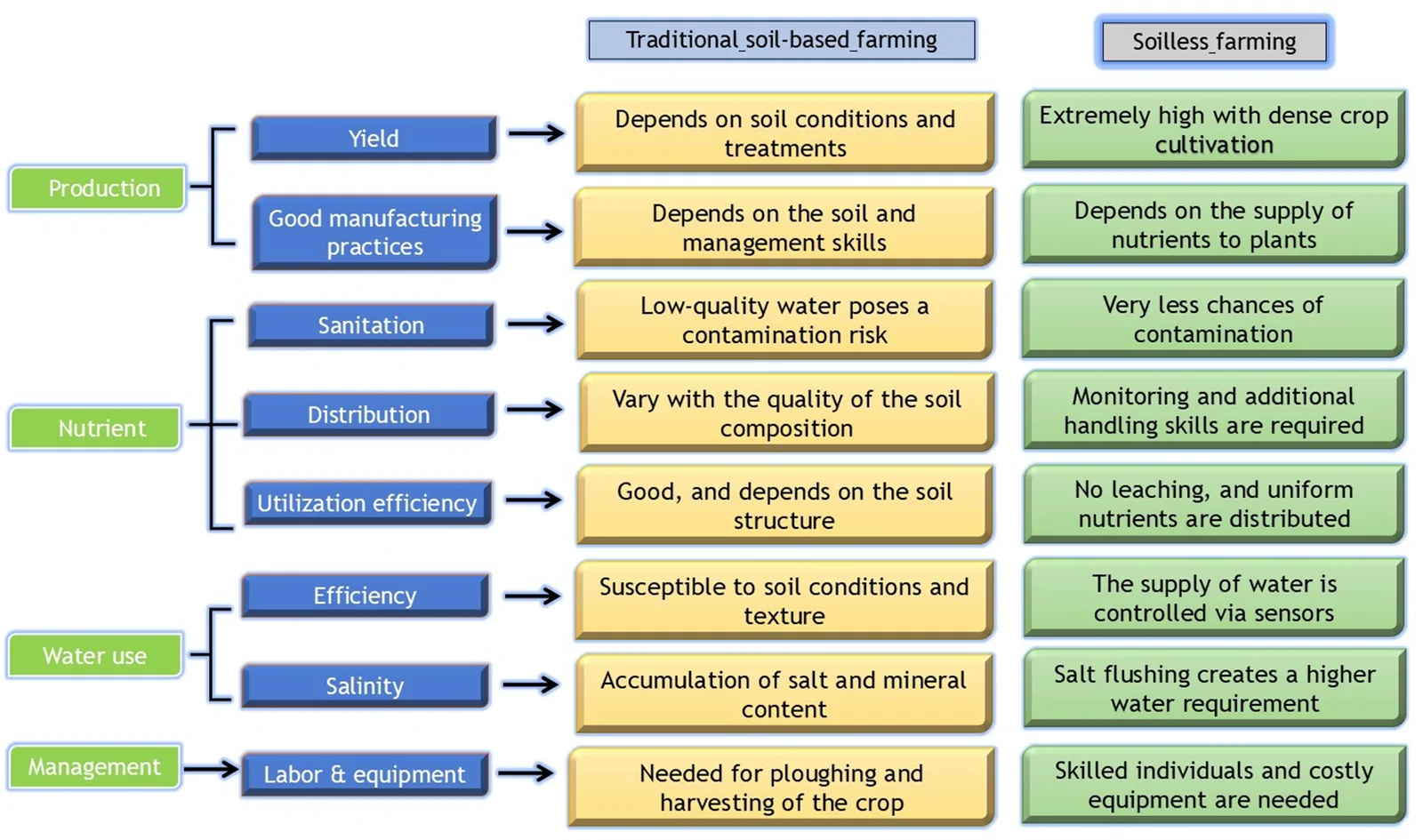
Comparison of soil-based and soilless farming regarding production, nutrients, water use, and resource management efficiency. Concept adopted from Wong et al., 2020.

Variations in LED light spectra, salinity stress, temperature, and nutrient availability can alter photosynthesis and respiration, modulate hormone signaling pathways, and ultimately affect the synthesis of phytochemicals (Paponov et al., 2023). The incorporation of elicitors into hydroponic systems is an effective approach to enhancing secondary metabolite production. Elicitors, which may be biotic (e.g., microbial extracts) or abiotic (e.g., yeast extract, methyl jasmonate), activate stress-like responses in plants under controlled conditions. This strategy effectively enhances the biosynthesis of high-value phytochemicals without detrimental effects on plant growth. Advances in genome editing offer opportunities to enhance resource use efficiency, refine desirable traits, and modulate plant height and stem length, ultimately improving plant architecture (Lim et al., 2025; Yu et al., 2025). Furthermore, climate-smart and controlled-environment agriculture leverage AI, IoT, ML, and automation to enhance productivity, sustainability, and precision in soilless farming (**Figure 2**). These technologies enable real-time monitoring, predictive modeling, and efficient resource management, thereby addressing the challenges of traditional farming. Although elevated expenses, technical requirements, and their integration present an appealing option for efficient food production and urban food security.

**Figure 2:**
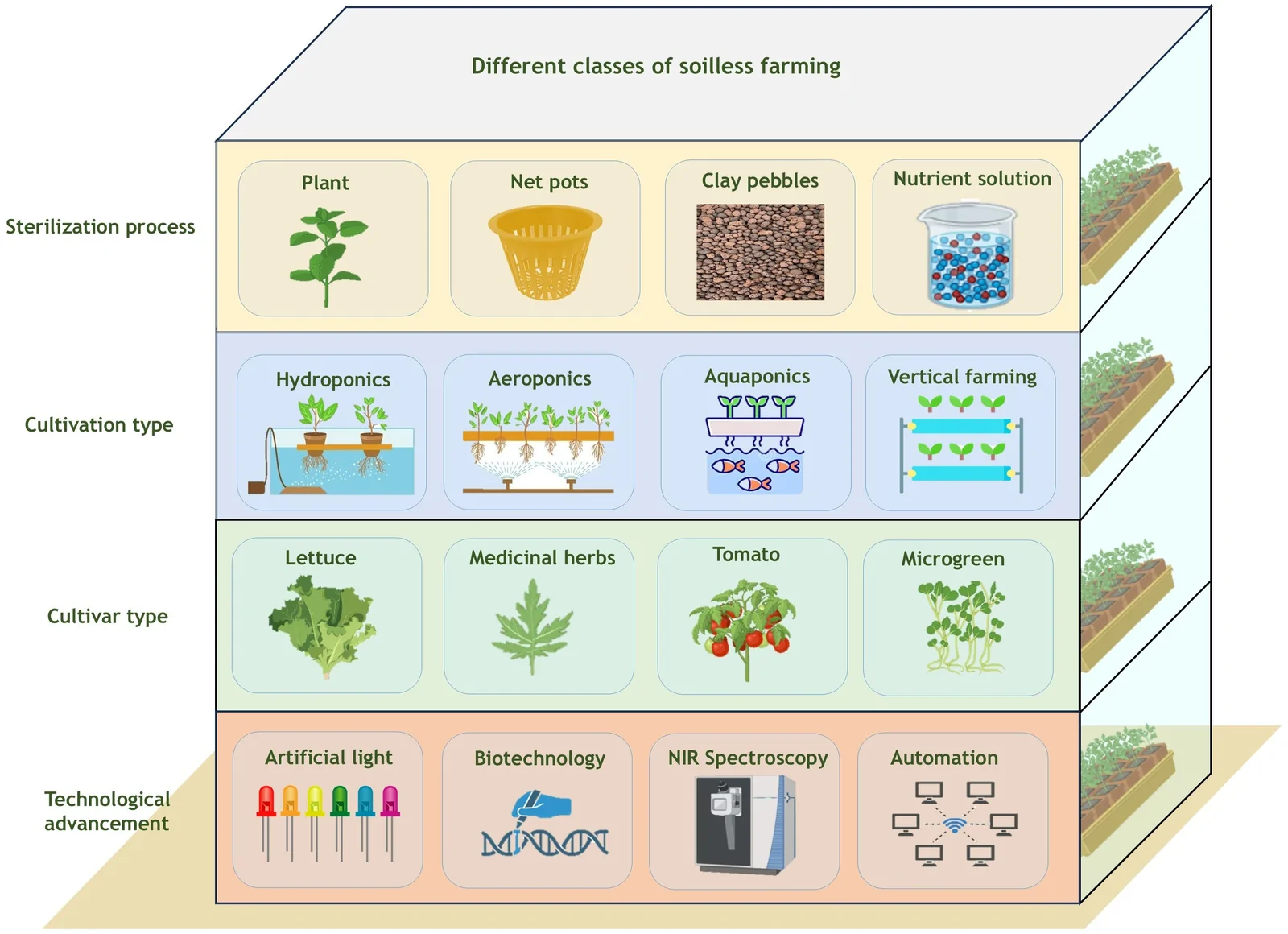
Schematic representation of different soilless cultivation systems, sterilization processes, cultivar types, and technological advancements. Concept adopted from Wong et al., 2020.

This review article offers a comprehensive evaluation of soilless cultivation strategies aimed at sustainable production of high-quality biomass and bioactive molecules, with a focus on medicinal plants. It analyzes various soilless cultivation systems, nutrient management strategies, dynamic LED lighting, and the integration of hydroponics with nanotechnology to optimize plant growth, photosynthesis, and phytochemical biosynthesis. The review also emphasizes the significance of precursor feeding, microorganism-based biostimulants, and elicitation strategies in enhancing biomass yield and specialized metabolite production. Furthermore, the review discusses the concept of reusable substrates in soilless farming to enhance its sustainability. Additionally, it discusses the emerging roles of computational technologies, and CRISPR/Cas-mediated plant architectural engineering as precision tools for improving crop productivity and metabolic outcomes. Together, these integrated approaches establish soilless agriculture as a promising platform for the standardized production of high-quality biomass and commercially valuable bioactive molecules, while enhancing resource-use efficiency and supporting sustainable medicinal plant cultivation.

## An overview of soilless farming

Soilless farming is a technique in which plants are grown without soil. Soilless farming encompasses various types of advances, including hydroponics, aeroponics, and aquaponics, alone or in combination. In these high-tech systems, plants recirculate, manage, and regulate the nutrient solution periodically (Kumar et al., 2024).

### Hydroponics cultivation

Hydroponics is a type of soilless cultivation in which plant roots are submerged/dipped in a liquid artificial medium (Sharma et al., 2024). In hydroponics, various substrates are used to support and grow plants, such as rock wool, gravel, pebbles, vermiculite, and perlite (Savvas and Gruda, 2018). In this system, liquid nutrients are supplied to the growing medium to provide the necessary nourishment, including micro- and macroelements, vitamins, and minerals, for optimal plant growth (Nguyen et al., 2016). Hydroponics is subdivided into many subclasses, such as nutrient film technique (NFT), wick system, deep water culture (DWC), ebb and flow (Flood and Drain), and drip feed system. This technique is commonly employed for cultivating leafy vegetables, culinary condiments, fragrant and medicinal plants, floral herbs, and fruit-bearing crops (Partap et al., 2020; Partap et al., 2022). Growers frequently assert that hydroponics consistently enables them to achieve higher productivity and production, unaffected by climate and weather constraints (Rajaseger et al., 2023). Furthermore, producers frequently assert that hydroponic food is of higher quality due to its use of a meticulously controlled environment, which facilitates a more uniform production process without waste of water and nutrients. Additionally, hydroponics is not reliant on seasonal variations, resulting in consistently high and uniform plant productivity.

### Aeroponics cultivation

In aeroponics, plants grow in a closed chamber, supported by artificial media, without the need for soil (Partap et al., 2020). This is a hydroponic farming practice in which the plant roots are suspended in a sealed container. The roots are kept in the dark while being exposed to a nutrient-rich water spray delivered via atomisers, a nozzle, and a fogger system (Lakhiar et al., 2018). Nevertheless, the practice of cultivating plants in the air using artificial support and atmosphere is very recent. The researchers conceived the notion of observing the predominant vegetation on rocks near the waterfalls. They often noted that plants thrived on rocks near waterfalls, with their roots exposed to air. It was the natural progression for cultivating plants in an environment where they were exposed to a fine mist of water. The solution spray periodically delivers nutrients to plant roots for a specific duration, rather than continuously misting (Lakhiar et al., 2018). Aeroponics technology is often effective for cultivating subterranean food storage crops such as potatoes and rhizomatous herbs (Partap et al., 2020).

### Aquaponics cultivation

Aquaponic systems combine fish farming with plant cultivation, establishing a symbiotic relationship in which fish waste supplies nutrients to plants, and plants help purify the water for fish (Kumar et al., 2024). Recirculating aquaculture systems are primarily designed to cultivate a large number of fish in limited water by purifying the water to remove harmful waste substances and then reusing it for fish farming (Okomoda et al., 2023; Kumar et al., 2024). Although nitrifying bacteria such as *Nitrosomonas* and *Nitrobacter* species convert harmful ammonia, nitrite, and phosphate into less harmful nitrate, a form of nitrogen that is readily available to plants (Goddek and Keesman, 2020). This closed-loop nutrient cycle reduces reliance on artificial fertilizers and minimizes nutrient release into the environment, thereby promoting sustainable nutrient management strategies. Hydroponics, such as DWC and NFT, are commonly utilized in aquaponics cultivation (Maharana and Koul, 2011; Okomoda et al., 2023).

## Nutrient management strategies in soilless farming

Nutrient solutions and their management are crucial for the performance of a soilless cultivation system. An optimum distribution of nutrients is essential for efficiently utilizing resources and maintaining solution solubility. The kind of fertilizers, feeding method, pH, electrical conductivity (EC), dissolved O_2_ concentration, root zone temperature (RZT), and management strategy are crucial factors that significantly impact plant growth, development, and the accumulation of bioactive substances in a soilless system (Al Meselmani, 2022; Kumar and Partap, 2026). The nutrient supply in the soilless system has a considerable impact on the nutrition, taste, texture, color, and other attributes of crops and plants (Levine and Mattson, 2021). Plant nutrient uptake occurs only when nutrients are in an absorbable form, typically ionic. For instance, nitrogen is taken up by plants as ammonium (NH_4_^+^) or nitrate (NO_3_^−^). *Echinacea purpurea* is cultivated hydroponically using varying concentrations of ammonia and nitrate (90:10 and 70:30). The study indicated that increasing the NO3^−^/NH4^+^ ratio significantly improved growth and antioxidant capacity (Ahmadi et al., 2021). The growth of plants is significantly influenced by pH. The reason is that nutrients supplied to the nutrient solution can only be dissolved in water and absorbed by the plant at specific pH values (Al Meselmani, 2022). The pH level is commonly regulated within the range of 5.5-6.5 in soilless farming, which has been observed to support optimal growth and nutrient absorption in nearly all cultivated crops (Partap et al., 2020). Nevertheless, the ideal pH for achieving the highest growth varies not only across species but also among cultivars, climatic conditions, and substrates. Frick and Mitchell (1993) suggested that the pH of a hydroponic nutrient medium varies due to imbalanced exchange interactions between roots and cations and anions. In vertical farming or hydroponics, the risk of exceeding or falling outside the optimal pH range increases when trying to maintain values near the edges. The constrained quantity of nutrients allocated to each plant’s roots increases its susceptibility to fluctuations. For example, increased pH promotes the precipitation of calcium and magnesium while diminishing the solubility of iron and phosphate in the solution. Concurrently, reduced pH levels impair the absorption of macronutrients, including nitrogen, calcium, and magnesium (Velazquez-Gonzalez et al., 2022). In *Araxacum officinale* and *Reichardia picroides*, a low pH (pH 4) enhances the synthesis of antioxidant molecules, improves photosynthesis, and overall plant development (Alexopoulos et al., 2021). The solution’s EC measures the salt concentration and indicates the level of electrolytes present. This measurement is directly related to the number of ions available to plants. The ideal EC range for most hydroponic crops is typically between 1.5 and 3.5 dS m^−1^, but this range may vary with crop type and developmental stage (Nguyen et al., 2022). Partap et al. (2020) suggested that the 2.0-2.8 mS cm^-1^ EC range was optimum to ensure good plant growth and secondary metabolites accumulation in aeroponically grown *Valeriana jatamansi*. In *Acmella oleracea*, EC of 3.5 mS cm^−1^ increases the essential oil content (do Carmo et al., 2024), and in *Centella asiatica*, EC 1.2 enhances the plant development and centellosides accumulation (Shawon et al., 2023). Apart from pH and EC, RZT also affect plant growth and physiology, including nutrient and water absorption (Thakur et al., 2019). Levine et al. (2023) suggested that the treatment at a RZT of 25 °C resulted in the highest dry weight of both shoots and roots, while 35 °C inhibited plant development but markedly enhanced anthocyanins and carotenoid content in Lettuce. Aidoo et al. (2019) demonstrated that exposing *Capsicum annuum* to RZTs of 7, 17, and 27 °C resulted in a decrease in shoot dry biomass at 7 °C compared to 17 °C and 27 °C in an aeroponic system. Root growth increases gradually with rising temperatures from the minimum to the ideal level; however, further increases in RZT lead to a rapid decline in both root and shoot growth (Yamori et al., 2022; Levine et al., 2023). The higher temperature may have enhanced mineral solubility and increased absorption, as the rate of solute dissolution tends to increase with temperature (Xu et al., 2006). Root nutrient absorption in most plant species is reduced under lower RZT conditions (Kang et al., 2025). The temperature of the root zone significantly affects the production of various plant metabolites across diverse plant types, including medicinal and culinary herbs (Lenni et al., 2020). Ensuring an adequate oxygen (O_2_) concentration in a nutrient medium in soilless farming is crucial for proper root aeration and nutrient absorption (Al Meselmani, 2022). In hydroponics, reduced O_2_ concentrations markedly affect root physiological activities, resulting in diminished respiration and water absorption (Chun and Takakura, 1994). Roosta (2024) suggested that low O_2_ concentrations (1.8 ± 0.2 mg. L^-1^) in nutrient solution led to a decrease in plant growth, while higher O_2_ concentrations (5.3 ± 0.2 mg. L^-1^) enhanced morphological growth and chlorophyll content in pepper plants in hydroponics. In a soilless culture, the roots may experience oxygen shortage if oxygen demand exceeds supply. This can lead to slower root growth and reduced uptake of ions and water. Plants cultivated in hydroponic systems can rapidly deplete the dissolved oxygen in the nutrient solution, leading to inadequate root aeration, particularly at elevated greenhouse temperatures. So, it is important to ensure adequate aeration of the nutrient medium to provide sufficient oxygen levels for plant root cells, which are essential for normal cellular activity and the microbial ecosystem (Al Meselmani, 2022).

## Artificial LED lighting technology for enhanced metabolites production

Plants cultivated in indoor systems are enclosed by walls and do not get natural sunlight; as a result, artificial lights serve as the sole source of light (Jung and Arar, 2023). Nevertheless, traditional light sources have disadvantages in their use, resulting in excessive heat on leaf surfaces and thus producing unfavorable consequences for plant growth. Hence, there is a need to create innovative artificial lighting sources to enhance lighting conditions, with significant breakthroughs offering unparalleled benefits (Erekath et al., 2024). Their use in plant cultivation illumination has experienced fast growth and enhanced crop yields (Jaeger, 2024). The emergence of light-emitting diodes (LEDs) for solid-state lighting in the early 1960s, coupled with technological and cost advancements, made LED lights economically viable for use in vertical farming (Pattison et al., 2018). LED lights offer advantages such as reduced electricity use, lower maintenance costs, and the ability to easily adapt lighting recipes for many crops (Paucek et al., 2023; Jaeger, 2024). The ability to regulate the wavelength and spectrum of LEDs enables optimization of light quality to precisely match the photosynthetic pigments and photoreceptors of crops, thereby enhancing efficiency. Light influences the production of secondary metabolites in plants by serving as both an energy source and an environmental signal. Plants sense different wavelengths of light using specific photoreceptors: phytochromes sense red and far-red light, cryptochromes and phototropins respond to blue light, and UVR8 detects UV-B. When these photoreceptors are activated, they trigger calcium influx, ROS production, and mitogen-activated protein kinase (MAPK) signaling. Furthermore, these downstream signaling control light-responsive transcription factors, associated genes, and hormonal crosstalk. Together, these networks regulate the activity of key genes and enzymes that drive the production of phenylpropanoids, flavonoids, terpenoids, and other specialized metabolites (Murthy et al., 2024; Kumar et al., 2026). As a result, changes in light quality and intensity can affect photosynthetic carbon supply, redox balance, hormone signaling, and metabolic flux, thereby influencing metabolite production. Using a customized spectrum consisting of wavelength combinations can accurately regulate plant metabolism, thereby increasing the synthesis of desired bioactive metabolites (Holopainen et al., 2018). The advancements in LED lighting technology, including the use of various colors and combinations of different light such as white light (WL), red light (RL), far-red light (Pfr), blue light (BL), purple light (PL), green light (GL), and yellow light (YL), have made it possible to modify the light spectra and intensity. This has facilitated research and development of light quality to improve the growth, color, flavor, biomass, secondary metabolite accumulation, and phytonutrient content of medicinal herbs under controlled environments (Dou et al., 2017; Neo et al., 2022). Partap et al. (2025) reported that a combination of BL and RL enhanced morphological traits and physiological growth, along with increased expression of flowering-related genes *FLOWERING LOCUS T* and *CONSTANS 9* and reduced expression of *the* flowering repressor *FLOWERING LOCUS C* gene in Lilium cultivated under the pin-trey soilless system. Paponov et al. (2023) reported that exposing the roots of *Artemisia annua* to WL, BL, and RL increases the production of artemisinin in the shoot tissue by 2.3-, 2.5-, and 1.9-fold, respectively, in aeroponic conditions. Similarly, in *Hypericum perforatum*, root exposure to WL, BL, and RL and GL increases the production of coumaroylquinic acid in leaves by 89, 65, 84, and 74%, respectively. *H. perforatum* grows hydroponically, and it has been reported that plant morphology, biomass, leaf area, and flower number showed a substantial increase when the RL/BL ratio was enhanced, particularly under the treatment of 100% RL. The flower achieved the highest levels of hypericin, pseudohypericin, and hyperforin when exposed to 100% RL (Karimi et al., 2022). Inthima and Supaibulwatana (2025) examine the effects of WL, RL, BL, and GL light on the growth and specific bacoside synthesis in diploid and tetraploid *Bacopa monnieri*. The study highlighted that BL enhances shoot length, while GL enhances dry biomass, production of specific bacosides (bacoside A3, bacopaside II, bacopaside X, and total bacoside), and gene expression levels (*FPS, bAS*, and *GT1*) in tetraploid *B. monnieri* plants. Light has become a focus for stimulating phytochemical production because it offers specific wavelengths, is cost-effective, and is long-lasting. Numerous studies have examined the effects and quality of various light sources on increased secondary metabolite synthesis in multiple plant species under indoor farming conditions (Karimi et al., 2022; Paponov et al., 2023). Many studies still struggle to accurately illustrate the mechanisms by which the production of pharmacologically significant secondary metabolites is stimulated. This is due to variations based on the specific plant species, growth phase, and light source employed. Consequently, the effective utilization of the light spectrum to improve or regulate the production of bioactive molecules requires further investigation to elucidate species-specific reactions (**Figure 3**).

**Figure 3:**
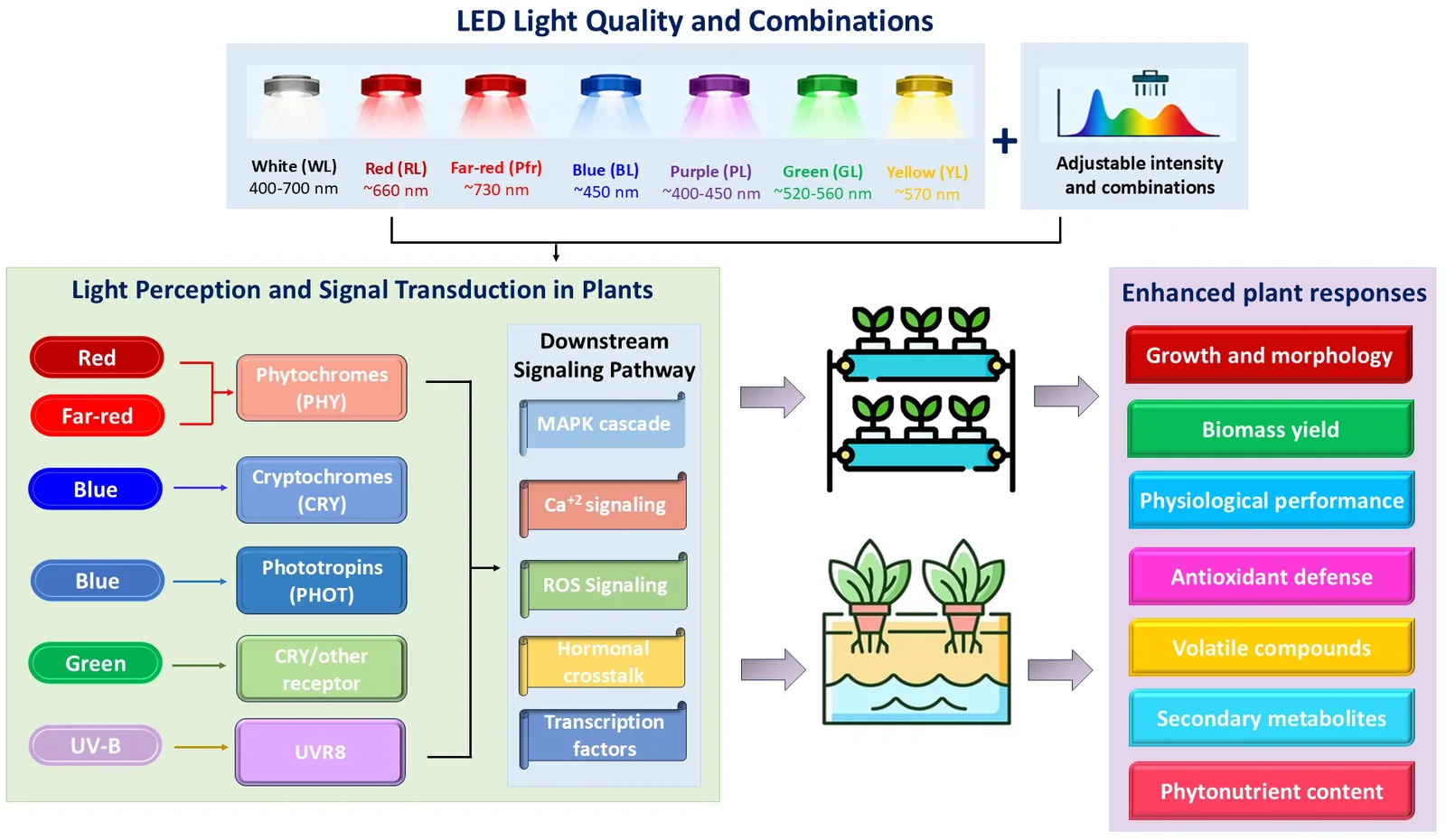
Schematic representation of LED light quality, photoreceptor perception, downstream signaling pathways, and enhanced physiological and metabolic responses in medicinal plants.

## Nano-elicitation for metabolite enhancement

Elicitation is a biochemical process that triggers plant defense mechanisms and stimulates the production of specific compounds in plant cells. This is achieved by activating genes that control the molecular pathways responsible for the synthesis of bioactive compounds in plants (Namdeo, 2007). Nanotechnology has evolved as a dynamic approach that has beneficially transformed numerous areas. Nanoparticles (NPs) have emerged as useful, innovative abiotic elicitors (Nourozi et al., 2019). The integration of nano-elicitation and soilless farming represents a promising cultivation strategy, offering precise nutrient management and enhancing plant productivity (Alikhani et al., 2021; Srivastava and Bhargava, 2022; Rani et al., 2025). Additionally, nano-elicitation enhances plant traits to withstand environmental diseases and stress, and the creation of field-sensing systems that monitor crop conditions and stressful effects, leading to increased production rates, crop yields, energy savings, and minimized waste (Marchiol, 2018; Jadoon et al., 2024). Soilless farming has gained popularity as a platform for studying the impact of NPs on plant growth and performance. NPs, with their small size (less than 100 nm) and large surface area, can enhance plants’ access to soluble, usable forms of nutrients and help prevent the precipitation and insolubilization of nutrients in the medium. The distinctive characteristics of NPs, such as their tunable physicochemical properties and capacity to penetrate plant cell walls, can enhance nutrient absorption by plant roots (Rani et al., 2025). NPs are efficient nutrient carriers for plants compared to conventional fertilizers, making them a highly promising tool, especially for soilless growing systems. Recent studies on NPs synthesized by environmentally friendly techniques have demonstrated their comparable efficacy to other substances in promoting plant development, increasing crop output, and boosting the content of phytochemicals. They enhance the synthesis of plant bioactive compounds, demonstrating their capacity to promote sustainable crop advancement. Hayat et al. (2021) demonstrated that AgNPs and CuNPs synthesized with *Moringa oleifera* extract significantly elevated the phenolic and flavonoid content in *Artemisia absinthium*, leading to an increase in antioxidant activity of up to 86%. The use of CuO and ZnO NPs improved antioxidant levels and pest resistance in *Amaranthus hybridus* (Francis et al., 2022). In *Silybum marianum*, Mubeen et al. (2022, 2023) recorded enhanced phenolic, flavonoid, and silymarin content, following treatment with AgNPs (1 ppm) along with *Aspergillus niger*, and MeJA as elicitors. Biologically produced silver and copper NPs enhance the levels of phenolics, flavonoids, alkaloids, and saponins in hydroponically cultivated *B. monnieri* (Lala, 2020). The feeding of magnetic NPs with salicylic acid elevates the concentration of phenolic, flavonoid, and aromatic content in *Calotropis procera* (Adabavazeh et al., 2022). The application of AgNPs in hydroponic cultivation enhanced carnosic acid, a bioactive compound known for its antibacterial and antioxidant properties, in rosemary plants (Soltanabad et al., 2020). Baharmoghadam et al. (2024) treated *Carum copticum* with varying concentrations of TiO_2_ NPs under different light conditions in hydroponics. The study found that TiO_2_ at 300 ppm and TiO_2_ NPs at 400 ppm, under white and blue light, significantly enhanced vegetative parameters, physiological growth, and essential oil content (α-pinene, γ-terpinene, and thymol). Abovementioned discussed investigations indicate that the combination of these two methods has significantly enhanced plant growth and the production of bioactive molecules (Mubeen et al., 2023; Jadoon et al., 2024). Nano-elicitation has been revealed to augment plant vitality and improve their defenses by enhancing the plant’s inherent defense mechanism. Consequently, integrating nano-elicitation with soilless agriculture has the potential to enhance sustainable farming practices and address the challenges posed by climate change and conventional farming methods.

## Vertical farming and hydroponic cultivation of medicinal plants

Most research indicates that plants grown using soilless cultivation, such as hydroponics, aeroponics, or vertical farming, can exhibit higher yields and greater concentrations of specialized phytochemicals (Kumar et al., 2025a; Rani et al., 2025). This may be associated with precise control of nutrient availability and EC, which generate mild nutritional or osmotic stress and stimulate the accumulation of stress-responsive metabolites (Jakovljevic et al., 2019; Partap et al., 2020). Mechanistically, controlled light, nutrients, pH, EC, dissolved oxygen, RZT and elicitor application, ROS, and phytohormone mediated signaling, thereby regulating photosynthesis, nutrient uptake, antioxidant defense and specialized metabolism pathways. These responses can direct metabolic flux toward phenolics, terpenoids, alkaloids, and other bioactive compounds. Soilless systems also facilitate the recovery of phytochemicals from plant tissues and root exudates, with exudates offering the advantage of non-destructive and potentially repeated harvesting throughout the plant life cycle (Partap et al., 2020; Rattan et al., 2022). Furthermore, it is necessary to effectively manage pH, EC, dissolved oxygen, nutrients, and RZT in the nutrient medium to ensure efficient nutrient uptake and enhance plant performance (Sharma et al., 2026). Continuous or periodic monitoring of nutrient solutions is essential to minimize nutrient imbalances and deficiency. Implementing effective sanitation and disinfection practices reduces the risks associated with pests and pathogens. Additionally, optimizing root fertigation, illumination, and elicitor application is necessary to maximize phytochemical accumulation in roots, shoots, and root exudates, while sustaining biomass productivity and product quality.

Multiple researchers have observed a notable increase in metabolite content in both horticultural and medicinal crops grown using hydroponics and aeroponics (Maggini et al., 2022; Rattan et al., 2022). A rise in the concentration of sesquiterpenes was observed in hydroponically grown *Chrysanthemum* (Yang et al., 2005). *V. officinalis* cultivated using the floating technique exhibited higher root dry weight than that of plants cultivated in a 1:1 perlite-vermiculite mixture (Tabatabaei, 2008). Xu et al. (2009) reported a new phytoconstituent, 2,3-Dihydrowithaferin A-3β-O-sulfate (a prodrug of withaferin A), discovered in *W. somnifera* cultivated aeroponically. Maggini et al. (2011) grew basil cultivars in hydroponics and reported elevated levels of echinacoside and rosmarinic acid. Bieberstein et al. (2014) conducted a study in which they grew *W. somnifera* using hydroponics and aeroponics to compare biomass and withaferin A content. The findings indicated that the hydroponically grown *W. somnifera* exhibited greater mean dry biomass and higher withaferin A levels compared with aeroponically cultivated plants. Thakur et al. (2019) demonstrated that *Picrorhiza kurroa* can be successfully cultivated both aeroponically and hydroponically; however, the aeroponic system was most effective and efficient for producing biomass and improving picrosides accumulation. Partap et al. (2020) examined the potential to increase yield and secondary metabolite synthesis in *V. jatamansi* under aeroponic conditions by employing yeast extract and MeJA as elicitors. Outcomes showed that both the elicitors enhance plant morphology, biomass, plant physiology, and metabolite accumulation (Isovaleric acid, patchouli alcohol, and baldrinal) under hydroponic conditions. Gangopadhyay et al. (2021) revealed that aeroponics offers significant benefits, including unrestricted access to plant roots without harming endangered species and promoting disease-free plants. The increased synthesis of 2-hydroxy-4-methoxybenzaldehyde in *Hemidesmus indicus* under aeroponic systems presents a new opportunity for the pharmaceutical and nutraceutical industries to utilize this approach for large-scale manufacture of endangered medicinal plants to meet the global and industry demand. Ahmadi et al. (2021) studied the impact of perlite and peat moss, along with variable ratios of NO_3_^−^/NH_4_^+^, on the phytochemical characteristics of the *Echinacea purpurea* in hydroponics. A reduction in perlite size and a growth in the NO_3_^−^/NH_4_^+^, ratio led to a notable rise in the phenolic compounds and various concentrations of caffeic acid derivatives, such as chicoric acid, caftaric acid, chlorogenic acid, cynarin, and echinacoside. Additionally, Zantanta et al. (2022) found that *Helichrysum odoratissimum* plants grown using aquaponics had the strongest antifungal properties. On the other hand, hydroponically cultivated plants had the highest levels of flavonols and antioxidant activity in their extracts. Mubeen et al. (2022) treated *Silybum marianum* with different elicitors (MeJA 100 mM) and (AgNPs 1 ppm) and found a significant increase in the accumulation of biochemical substances. Treatment with MeJA (100 mM) yields the highest levels of flavonoids, phenolics, and superoxide dismutase activity in hydroponic cultivation. In *Houttuynia cordata*, Yin et al. (2024) demonstrate that the hydroponic system offers a sustainable alternative cultivation to conventional agricultural practices by significantly diminishing the accumulation of hazardous benzene compounds. Multi-omics analysis revealed that, in hydroponics, enhanced nitrogen metabolism improves the expression of photosynthesis-related genes (*HcatpB, HcpetC*, and *HcpetH*), thereby considerably boosting biomass production. In *B. monnieri*, 50 µM salicylic acid significantly enhances plant morphological parameters, such as plant height, number of shootlets, and dry biomass, along with primary and secondary metabolites (geniposidic acid) (Vinay et al., 2025). In hydroponics and aquaponics systems, we observed a 23.6% and 60.4% increase in β-Myrcene, a 21.3% and 15.1% increase in geranial, a 41.9% and 31.1% increase in geraniol, a 78.9% and 60.6% increase in geranyl acetate, and a 78.8% and 60.6% increase in juniper camphor, respectively, compared to the soil system in lemongrass (*Cymbopogon citratus*) (Alizadeh et al., 2025). Similarly, Agrahari et al. (2025) cultivated gray water-treated *W. somnifera* in hydroponics and found that plants showed significant improvements in plant morphology, antioxidant activity, and chlorophyll content. Additionally, Giri et al. (2025) cultivated *Rheum tibeticum* under hydroponic conditions and found that it enhanced plant morphological growth, biomass accumulation, photosynthetic and antioxidant activities, and secondary metabolites production. Collectively, these studies suggest that integrating controlled environmental cues with fertigation and elicitation modulates plant physiological and metabolic responses, offering a sustainable strategy to enhance biomass production and phytochemical yield in medicinal plants. Different medicinal plant cultivation techniques under different soilless conditions are presented in **Table 1**.

**Table 1:** Medicinal plant cultivation in different types of soilless farming.

Medicinal plants	Secondary metabolites	Type of system	Cultivation condition	Part used	Type of response	References
*Withania somnifera*	Withanolides, Withaferin-A, and Withanone	Hydroponic	Temperature: 25˚C Photoperiod: 16 h light/8 h dark Relative humidity: Not controlled pH: 6.8–7.0 EC: 0.5-1.5 mS cm^−1^	Leaf Root	Withanone/Withaferin A rich leaves could be obtained by manipulating light conditions in hydroponic cultivation	Kaul et al. (2017)
*Valeriana jatamansi*	Valerenic acid, acetoxy valerenic acid, and hydroxy valerenic acid	Aeroponics	Temperature: 25 °C Photoperiod: 16 h light/8 h dark Relative humidity: 65% pH: 6.8–7.0 EC: 0.5-1.5 mS cm^−1^	Leaf Root	Elicitors (methyl jasmonate and yeast extract) promote plant growth and secondary metabolite content	Partap et al. (2020)
*Picrorhiza kurroa*	Picrosides (I, II, and III)	Aeroponics and hydroponics	Temperature: 25 °C Photoperiod: 16 h light/8 h dark Relative humidity: 65% pH: 6.8–7.0 EC: 0.5-1.5 mS cm^−1^	Leaf Stem Root Rhizome	Boost plant productivity and picrosides accumulation	Thakur et al. (2019)
*Silybum marianum*	Silybin A, silybin B, isosilybin A, isosilybin B, silychristin, silydianin, apigenin 7-D glucose, and taxifolin	Hydroponics	–	Leaf	Treatment with methyl jasmonate (100 µM) exhibited the highest flavonoid (304 µg g^−1^), phenolic (372 µg g^−1^), and superoxide dismutase (16.2 U g^−1^) content	Mubeen et al. (2022)
*Cichorium intybus, Withania coagulans*, and Echinacea	Bitter-tasting sesquiterpene lactones Withanolides and Withaferin Alkamides, polysaccharides, caffeic acid derivatives, and glycoproteins	Aeroponics	Temperature: 25 °C Photoperiod: 16 h light/8 h dark Relative humidity: 65% pH: 5.8 ± 0.2 EC: 1.6 ± 0.2 dS/m		Results in increased plant biomass and photosynthetic pigments as compared to the soil system	Movahedi and Rostami (2020)
*Crocus sativus*	Crocin, crocetin, and picrocrocin	Aeroponics, hydroponics, and soil	Temperature: 17 ± 2 °C Photoperiod: 11 h light/13 h dark Relative humidity: 65% pH: 6.0–6.25 EC: 0.5-1.0 mS cm^−1^	Stigma	Growth analysis and pigment content were found same in all 3 types of cultivation	Souret and Weathers (2000)
*Valeriana officinalis*	Essential oil content (bornyl acetate, valerenal, camphene, trans-caryophyllene, cis-ocimene, α-fenchen, and δ-elemene)	Aeroponic and soil cultivation	Temperature: 28 ± 3 °C day and 20 ± 3 °C night Photoperiod: 11 h light/13 h dark Relative humidity: 65% pH: 7.2 EC: 2.4 dS m^−1^		Aeroponics cultivation enhances essential oil concentration	Tabatabaei (2008)
*Stevia rebaudiana*	Steviol glycosides	Hydroponics and soil system	Temperature: 28 ± 3 °C day and 20 ± 3 °C night Photoperiod: 11 h light/13 h dark Relative humidity: 65% pH: 5.5 - 6.5 EC: 0.75 - 1.5 dS m^−1^	Leaf	Optimized nutrient concentration and growth media for stevia cultivation	Janakiramudu et al. (2022)
*Glycyrrhiza uralensis*	Glycyrrhizin	Hydroponic	Light intensity: 300 μmol m^−2^ s^−1^; relative humidity: 65–70%; CO_2_ concentration: 1000 μmol mol^−1^; air temperature: 28/26 °C.	Root extracts	Enhanced biomass production and elevated glycyrrhizin biosynthesis	Afreen et al. (2005)
*Rheum tibeticum*	Anthraquinones	Hydroponic	Electrical conductivity of 2.13 mS cm^−1^ and a pH of 6.15	Roots	Exhibited enhanced root development and increased below-ground biomass compared to soil-grown plants	Giri et al. (2025)
*Momoradica charantia*	Momordicosides	Hydroponic	Temperature 24 °C ± 4, relative humidity 50%±5	Roots, Leaves, Fruits	Double-strength Hoagland’s solution most effectively enhanced all morphological traits and reduced time to flowering and fruit harvest	Majidi et al. (2025)
*Nasturtium officinale*	Glucosinolates	Aquaponic, Hydroponic	The pH of the hydroponic solution was kept between 6.5 and 7.5, and EC (dS m^−1^) 2.83	Leaves	Enhanced morphological and physiological growth	Alizaeh et al. (2025)
*Glycyrrhiza glabra L.*	Glycyrrhizic acid	Aeroponic, nutrient film technique, Substrate-based system and soil system	Nutrient solution adjusted to pH 6.5 in the aeroponic system and EC (dS m^−1^) 1.69 and pH 7.2 of the soil used in this experiment	Roots	Ammonium nutrition enhanced root glycyrrhizic acid accumulation by 65%, 35%, and 44% in aeroponic, NFT, and neutral-substrate systems, respectively, with no significant difference between nitrate and ammonium nitrate treatments	Roosta et al. (2026)
*Ocimum basilicum*	Estragole (methyl chavicol), δ-cadinene, methyl eugenol, linalool, and β-farnesene	Hydroponic system	The pH of the hydroponic solution was adjusted to 6.2, temperature 22 ± 2 °C and relative humidity of 60 ± 5%	Aerial parts	Plant Growth-Promoting Microorganisms Phosphorus interaction (PGPM–P) treatments enhanced EO content from 0.1% to 0.4%, with increases in major metabolites including estragole (43.7%), δ-cadinene (16.9%), and methyl eugenol (10.7%).	Moharrami et al. (2026)

## Plant growth-promoting microorganism in soilless farming

Plants in their natural environment live alongside various microbial communities that can be beneficial, commensal, or pathogenic. Plants communicate with a variety of microorganisms in three primary areas: the phyllosphere, endosphere, and rhizosphere. Plant growth-promoting microorganisms (PGPMs) are beneficial microorganisms that inhabit plant rhizospheres and play a vital role in enhancing plant growth and development (Dhawi, 2023; Chiaranunt and White, 2023). In soilless farming systems, PGPMs are closely associated with plant roots and facilitate growth through several interconnected mechanisms. After colonizing the root zone, PGPMs improve nutrient uptake by enabling biological nitrogen fixation and solubilizing phosphate and other essential nutrients (Kolega et al., 2020). Siderophore production enhances iron uptake, while microbial synthesis or modulation of phytohormones such as indole-3-acetic acid, cytokinins, and gibberellins stimulates root development and nutrient absorption. Microbial exopolysaccharides and biofilm formation further support root-microbe associations and promote effective microbial colonization. These interactions can induce systemic resistance and activate antioxidant defense pathways via microbial elicitors and volatile compounds, thereby enhancing ROS scavenging and increasing tolerance to salinity, nutrient imbalances, and other environmental stresses (Moncada et al., 2020). As a result, PGPM-driven enhancements in nutrient uptake, hormonal balance, stress resilience, and antioxidant capacity contribute to increase biomass production and stimulate secondary metabolite biosynthesis (Zapata-Vahos et al., 2020; Mubeen et al., 2022, 2023; Dhawi, 2023). Therefore, PGPMs serve as versatile biological agents in soilless systems, enhancing plant growth, nutrient use efficiency, phytochemical synthesis, and resilience to environmental challenges and climate variability. The intricate and ever-changing relationships between plants and their microbiomes are shaped by the host, microbes, and environment, forming a complex triad that determines their ultimate outcome (Ali et al., 2022; Gerrewey et al., 2024). Various parameters in soilless farming may affect interactions between plants and PGPMs. The factors that influence this include the type of growing substrate, oxygenation, RZTs, the quality and intensity of light, the addition of CO_2,_ and the plant’s age (Chiaranunt and White, 2023). An AMF *Rhizophagus irregularis* in *Anchusa officinalis* enhances primary and secondary metabolites accumulation in root exudate (Cartabia et al., 2021). Fungal elicitor *A. niger*, along with MeJA, silver NPs, enhance antioxidant activity, silymarin and Silymarin flavonolignans content in *Silybum marianum* (Mubeen et al., 2022, 2023). In *Rhinacanthus nasutus, Trichoderma harzianum* (1.0 mg/mL) boosts rhinacanthin content (Suksawat and Panichayupakaranant, 2024). Replacing toxic pesticides with PGPMs in soilless farming is regarded as a highly effective and environmentally safe biotechnology strategy. Utilizing PGPMs in soilless farming holds the capacity to enhance crop productivity, promote ecological sustainability, and augment food security (Stegelmeier et al., 2022; Dhawi, 2023). Employing PGPM-assisted methods can contribute significantly to establishing a more robust agricultural model (Azizoglu et al., 2021; Mourouzidou et al., 2023; Hasan et al., 2024). Nevertheless, the primary inquiry persists: How can these instruments be efficiently utilized to devise a sustainable and efficient system? Although there is currently significant evidence demonstrating the potential of PGPM to decrease the use of fertilizers and develop efficient plant protection methods, additional research is required to comprehensively comprehend how to optimize the advantages of sustainable agriculture without compromising crop output (Mourouzidou et al., 2023; Hasan et al., 2024). **Figure 4** represents the role and importance of PGPMs in enhancing plant productivity and pathogen resistance in soilless farming.

**Figure 4:**
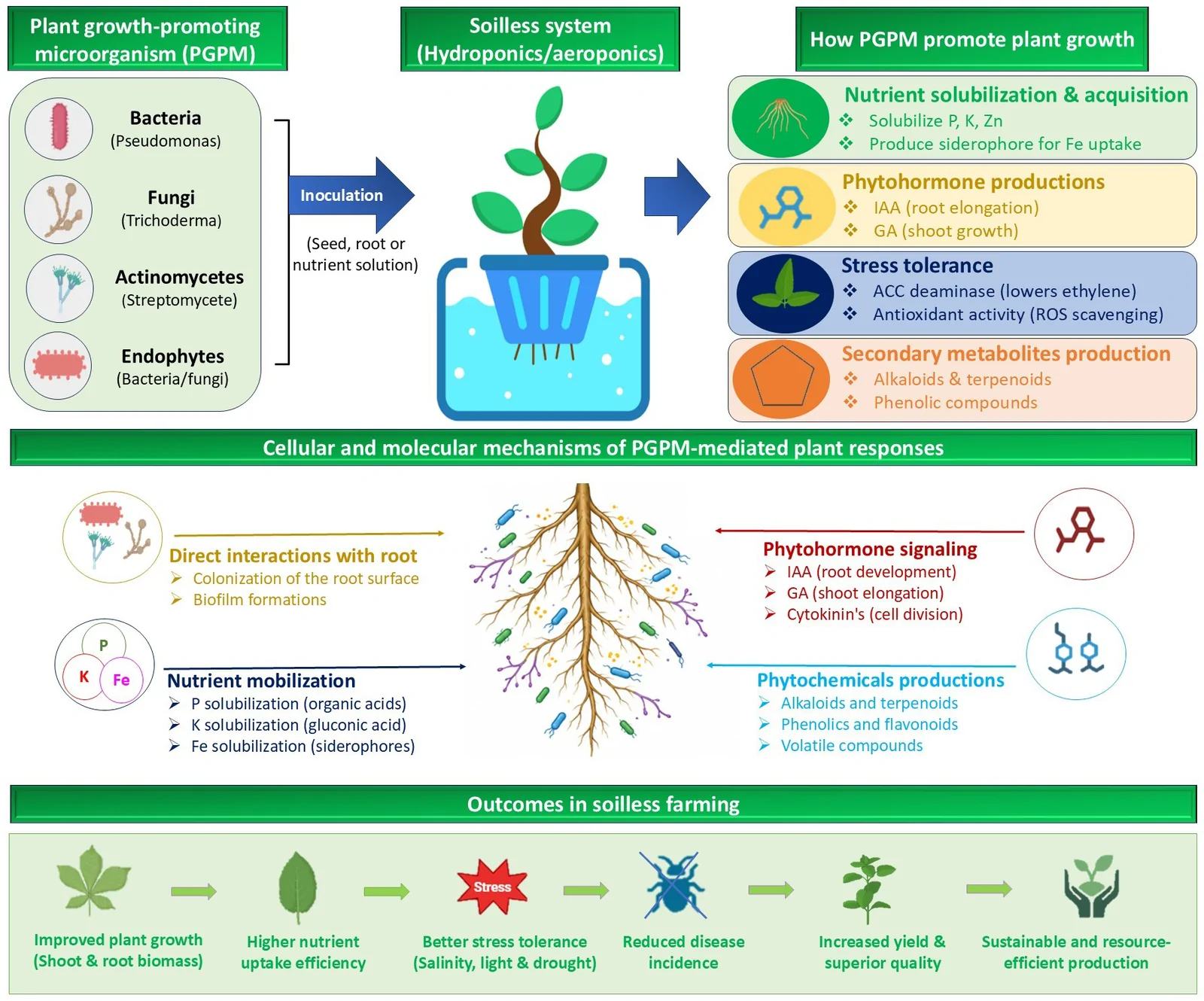
Mechanistic framework illustrating the role of plant growth-promoting microorganisms (PGPMs) in soilless farming, including nutrient mobilization, phytohormone production, stress tolerance, biocontrol, and root-system enhancement, leading to improved plant growth, yield, quality, and resource-use efficiency.

## Computational technologies and AI-driven precision management in soilless farming

Climate-smart agriculture and controlled-environment agriculture are innovative advances in agricultural engineering and biotechnology with the potential to meet future food and market demands (Gryshova et al., 2024). In this context, indoor and soilless farming systems provide a highly controllable environment in which cutting-edge computational technologies such as AI, IoT, robotics, machine learning (ML), and deep learning (DL) can be integrated for real-time data acquisition, analysis, predictions, and automated decision making (Jaiswal et al., 2024; Rajendiran and Rethnaraj, 2024; Erekath et al., 2024). Computational technologies are therefore emerging as important drivers of the digitization of smart agriculture (Petropoulou et al., 2023), enabling the transition of soilless production from conventional environmental monitoring toward data-driven precision cultivation. The integration of AI, IoT, and ML/DL into soilless systems enables continuous monitoring and optimization of critical environmental and nutritional parameters; therefore, such systems can facilitate precise resource management and improve the environmental sustainability and resilience of controlled agricultural production (Krishnan and Swarna, 2020). Intelligent decision-support systems reduce the need for human intervention and minimize operational errors, thereby enhancing the efficiency of indoor cultivation (Tik et al., 2009). Computational algorithms are increasingly utilized in agriculture for crop yield prediction, disease detection, environmental forecasting, water management, fertilizer optimization, and automated crop production (Siregar et al., 2022; Devi et al., 2026). In soilless farming, these technologies are especially significant because environmental and nutritional variables can be controlled with greater precision than in conventional systems. This precision provides a robust platform for predictive and closed-loop cultivation. Schematic representation of automation technology in soilless farming (**Figure 5**).

**Figure 5:**
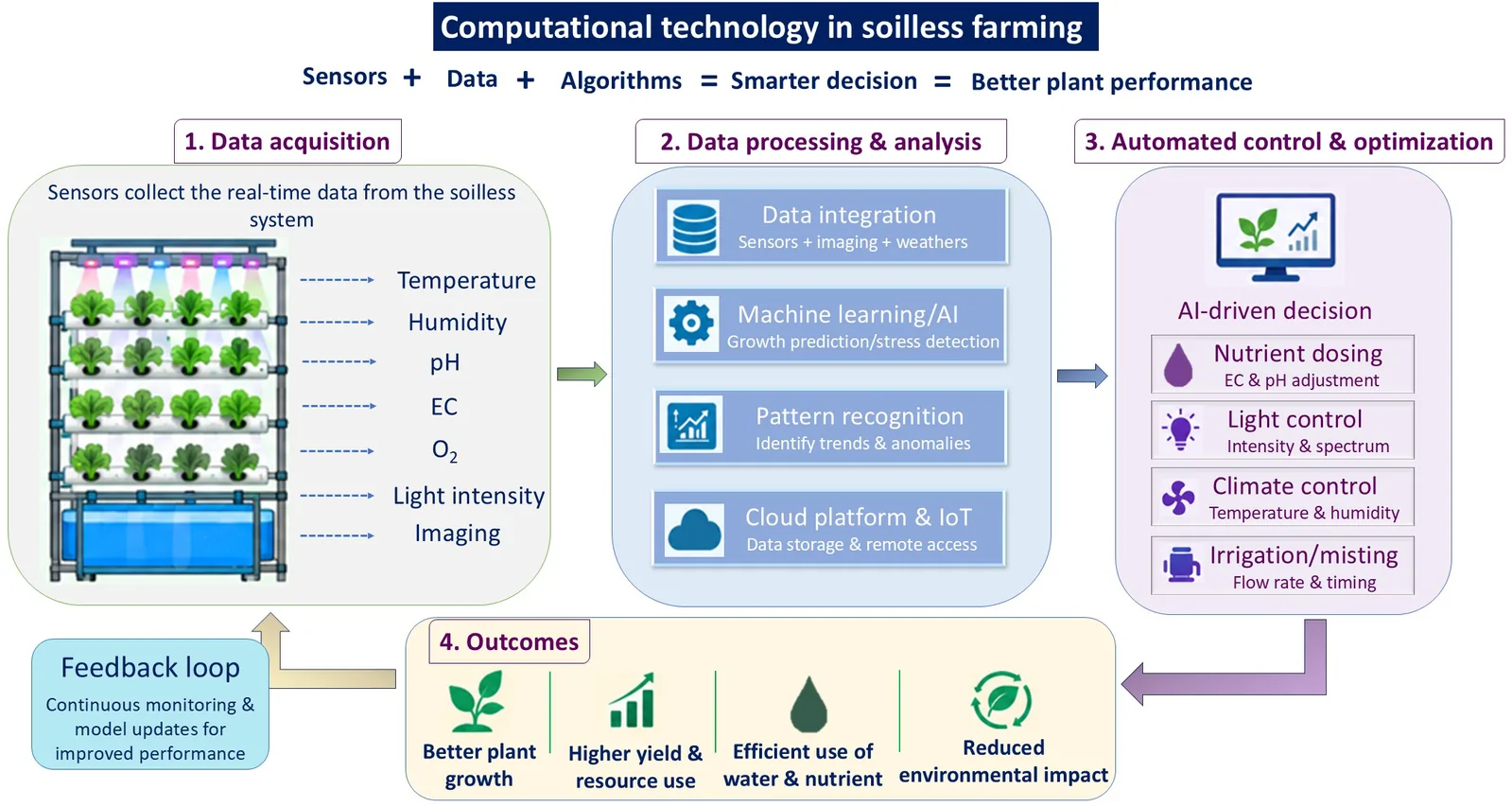
Computational technology-driven framework for smart soilless farming, integrating sensor-based data acquisition, AI/ML-assisted analysis, automated control, and feedback-driven optimization to improve plant growth, yield, resource-use efficiency, and sustainability.

Several studies demonstrate the feasibility of computational approaches in hydroponic and vertical farming systems. Huang et al. (2024) developed a growth-response model (GRM) that is highly effective at predicting leafy vegetable shoot biomass in controlled-environment vertical farms, demonstrating the potential of predictive modeling for crop growth management. Abukhader and Kakoore (2021) employed a genetic algorithm and a Job-Shop scheduling model to improve fruit-crop productivity and to anticipate the optimal harvesting time in an indoor vertical farming. Similarly, Rabka et al. (2021) developed a cost-effective, energy-efficient solar power method for remotely monitoring greenhouse farms. This method uses sensors to monitor soil moisture, CO_2_ levels, light intensity, temperature, humidity, and pressure in a hydroponic system. Recently, ML approaches have been applied to hydroponic nutrient and water management. Dhal et al. (2024) demonstrated that ML models can analyze nutrient and water uptake in hydroponically cultivated soybean, with Random Forest algorithms providing accurate predictions of water uptake. These methods show that sensor-derived datasets can be converted into predictive information to optimize resource application, rather than relying exclusively on fixed irrigation or nutrient schedules. In addition to the benefits of automation and computational technology in soilless farming, there are drawbacks, including costly infrastructure requirements, the need for technical expertise, and reliance on artificial inputs. Pros and cons of automation and computational technology in soilless farming (**Figure 6**).

**Figure 6:**
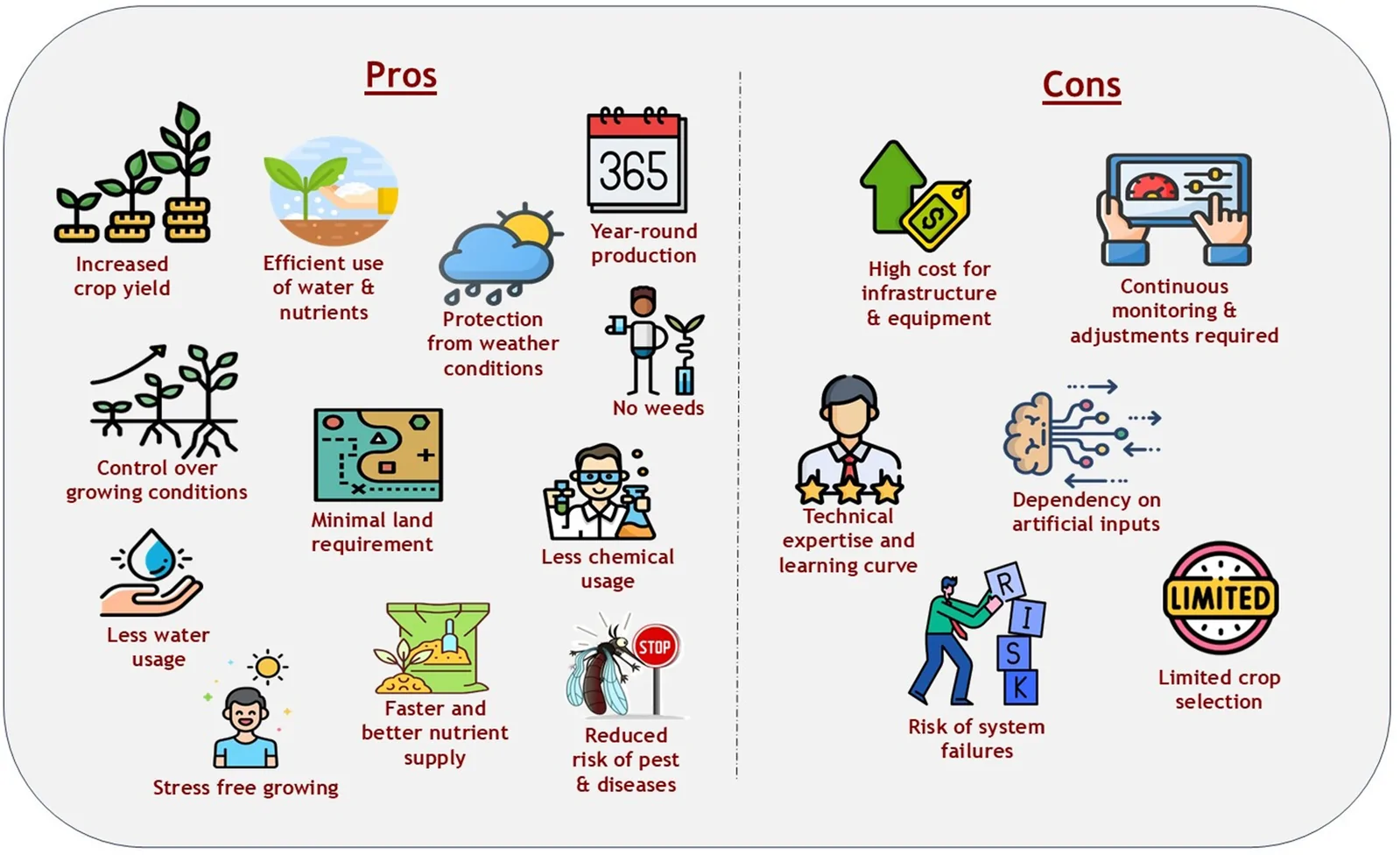
Pros and cons of automation and computational technology in soilless farming.

The integration of AI with advanced sensing technologies significantly enhances the potential for precision soilless farming. Computer vision and hyperspectral imaging enable non-destructive assessment of plant architecture, biomass, physiological status, and nutrient-related stress. Recent studies have combined hyperspectral imaging with ensemble ML algorithms to enable early detection of nutrient deficiencies in hydroponically cultivated crops, highlighting the capacity of AI-assisted imaging to identify nutritional stress before visible symptoms appear (Nagarajan et al., 2025). Additionally, ML-based growth prediction and anomaly detection systems have been implemented using environmental sensors and imaging data in hydroponic lettuce production (Métwalli et al., 2025). Collectively, these advancements represent a transition from traditional sensor-based monitoring to plant-centered sensing, where the crop itself serves as a continuous source of phenotypic and physiological data.

A significant emerging field in medicinal plant research involves the use of computational technologies not only for biomass optimization but also for predicting and regulating specialized metabolite biosynthesis. Medicinal plants are cultivated predominantly for specific phytochemicals such as phenolics, flavonoids, terpenoids, and iridoids rather than for biomass. To address this, future smart soilless systems should combine data from environmental, physiological, phenotypic, transcriptomic, and metabolomic sources to better understand how growing conditions affect phytochemical content. Studies using phenomics and metabolomics have already shown that certain environmental factors are associated with changes in specialized metabolites in hydroponically grown medicinal and aromatic plants (Ćavar Zeljković et al., 2022). Similarly, combining physiological and multi-omics data has helped reveal how growing conditions, basic metabolism, gene expression, and bioactive compounds are connected. These methods form the groundwork for AI models that can predict how phytochemicals respond to changes in light, nutrients, elicitation, temperature, and other environmental cues. Advancement of this concept is possible through the integration of AI with digital twins and closed-loop control systems. A digital twin of a soilless cultivation unit can continuously assimilate sensor, imaging, growth, and biochemical data to simulate plant responses across various cultivation scenarios. ML/DL models can predict the effects of adjustments to light quality, nutrient concentration, irrigation, temperature, or elicitor application, thereby enabling automated systems to implement targeted interventions in real time. AI-guided cultivation can therefore facilitate the simultaneous optimization of biomass, resource-use efficiency, and phytochemical yield. Future research should prioritize the development of standardized multi-year datasets, explainable and transferable AI models, integration of multi-omics and phenomics data, implementation of digital twins, and independent experimental validation of AI-driven modules. The integration of AI, ML, IoT technologies, advanced imaging, digital twins, and multi-omics approaches could facilitate real-time optimization of plant growth and specialized metabolite production while enhancing the efficiency of water, nutrient, and energy use. This transition has the potential to establish soilless farming as a highly controllable platform for the sustainable and targeted production of high-value phytochemicals. **Table 2** presents technological advancements in soilless farming aimed at improving plant productivity.

**Table 2:** Technological advancement in soilless farming for enhanced phytochemicals production.

Plant name	Used for	Technology used	System used	References
LED lighting technology				
*Artemisia annua* and *Hypericum perforatum*	Long-term exposure of roots to LED lighting of different wavelengths on the growth and phytochemical composition of two high-value medicinal plants, *Artemisia annua* and *Hypericum perforatum*	LED lights	Aeroponic	Paponov et al. (2023)
*Hypericum perforatum*	Biomass accumulation of stem, leaf, and root increased linearly with the increase of photosynthetic photon flux density (PPFD) spectrum	light intensity and LED spectrum	Hydroponic	Gao et al. (2022)
Basil (*Ocimum* sp.)	Blue (435 nm) and red (663 nm) light at a 1.6:1 ratio and 300 µmol m^-^² s^-^¹ intensity enhanced yield and growth	LED spectrum	Hydroponic	Aldarkazali (2020)
*Crocus sativus*	A strong correlation was found between light intensity, dry stigma yield, and levels of crocin, picrocrocin, and safranal, all of which significantly increased as artificial light rose from 2 to 8 mol·m^-^²·d^-^¹	Light intensity	Hydroponic	Farahani et al. (2025)
Nanotechnology				
*Fenugreek*	AgNPs promote the accumulation of phytochemicals, enhance nutrient uptake, and support overall plant growth	Nanoparticles Using *Bacillus safensis*	Hydroponic	Jadoon et al. (2025)
*Valeriana officinalis, Cichorium intybus, Withania coagulans* and *Echinacea purpurea*	Samples of valerian (*Valeriana officinalis* L.), chicory (*Cichorium intybus* L.), withania (*Withania coagulans*) and purple coneflower (*Echinacea purpurea* L.) were evaluated under aeroponic system conditions to explore the effects of zinc oxide nanoparticles	Nanotechnology	Aeroponic	Mohit Rabary et al. (2022)
*Silybum marianum*	AgNPs enrichment enhances secondary metabolites content	Nanotechnology	Hydroponic	Mubeen et al. (2022)
*Bacopa monnieri*	AgNPs treatment increases protein and carbohydrate content but reduces total phenolic content (TPC), catalase and peroxidase activity	Nanotechnology	Hydroponic	Krishnaraj et al. (2012)
*Carum copticum*	TiO_2_ NPs (300ppm) and light spectrum substantially boost anthocyanin and total phenolic content (TPC) in shoot tissue	Nanotechnology	Hydroponic	Baharmoghadam et al. (2024)
Biotic and abiotic elicitors				
*Bacopa monnieri*	Elevated iridoid monoterpenoids (aucubin, geniposidic acid) content	Salicylic acid	Hydroponic	Vinay et al. (2025)
*Valeriana officinalis*	Improved root length, dry weight, and biomass	Naphthalene acetic acid	Aeroponic	Rostami and Movahedi (2016)
*Hippeastrum papilio*	Salicylic acid (100 μM/L) concentration increases galanthamine content	Salicylic acid	Hydroponic	Haist et al. (2025)
*Silybum marianum*	5 days of treatment with salicylic acid (200 mM) enhances silymarin accumulation	Salicylic acid	Hydroponic	Ahmed et al. (2020)
*Agastache rugosa*	MeJA and nutrient solutions EC enhance tilianin and acacetin content	Methyl jasmonate and EC	Hydroponic	Kim et al. (2018)
Artificial intelligence technology				
*Pana*x *ginseng*	A hierarchical machine learning algorithm (artificial neural networks, support vector machines, and random forests) can predict germination and rottenness in ginseng sprouts	Machine learning approach	Hydroponic	Kim et al. (2023)

## Reusable substrates for sustainable soilless farming

The sustainability of soilless farming must encompass not only water and nutrient use efficiency but also the life cycle, durability, and post-use management of growing substrates. Conventional substrates, including peat and perlite, are typically used for a limited number of production cycles, leading to increased material demand and associated environmental disposal problems. Life-cycle assessments demonstrate that the environmental performance of growing media depends on factors such as material origin, processing, transportation, and end-of-life management. Consequently, substrate selection requires evaluation from a whole-system perspective rather than focusing solely on crop performance (Paoli et al., 2022; Alejandre et al., 2026). Therefore, Substrate reuse and its management constitute a significant and emerging aspect of sustainable soilless farming. Recent studies have demonstrated the feasibility of reusing growing substrates without compromising crop productivity and yield. For example, reused coir-based substrates previously used for spinach cultivation supported lettuce production, with yields comparable to those obtained with new coir, and some reused mixtures produced equal or higher concentrations of phenolics and ascorbic acid (Machado et al., 2023). These findings are especially relevant for medicinal and functional crops, as substrate reuse must be assessed for biomass production, accumulation of specialized metabolites, and overall product quality.

Researchers have also shown that spent peat- and perlite-based growing media can be reused in chrysanthemum production after steam treatment. Steam treatment significantly reduced phytosanitary risks, and the reused substrates maintained adequate functionality for subsequent cultivation. However, the study also found that reused media often contain significant residual nutrients, especially phosphorus and potassium, so fertilizer inputs should be adjusted accordingly (Vandecasteele et al., 2020). This observation reveals a significant research gap: effective substrate reuse requires not only repeated use of the material but also systematic characterization and management of the evolving chemical, physical, and biological properties in the root-zone environment. Findings from repeated peat reuse indicate that direct reuse is technically feasible across multiple production cycles. In commercial-scale trials, peat-based growing media reused for strawberry cultivation produced yields comparable to those of virgin substrate. Furthermore, repeated reuse over three cycles did not result in significant deterioration of physical properties or crop performance (Vandecasteele et al., 2024).

Despite its potential benefits, substrate reuse is associated with several challenges. The accumulation of salts, residual nutrients, and pathogens, as well as changes in microbial communities, can significantly modify the root-zone environment over successive cultivation cycles. Consequently, future research should establish standardized criteria, including water-holding capacity, porosity, and nutrient retention, to evaluate the suitability of substrates for reuse. Although interest in sustainable growing media is increasing, most research has focused on identifying alternative substrate materials rather than developing integrated strategies for repeated substrate recovery, sanitation, root-zone monitoring, and maintenance of phytochemical quality across multiple cultivation cycles. Therefore, advancing reusable substrates, combined with precision root-zone management and life-cycle assessment, constitutes a critical research direction for promoting sustainable soilless farming cultivation.

## Precursor feeding approach to boost phytochemicals production

Precursor feeding is a targeted metabolic engineering strategy in which pathway-specific substrates or intermediates are supplied exogenously to enhance the biosynthesis of desired compounds (Isah et al., 2018; Rattan and Warghat, 2023). The increased availability of precursors enhances metabolic flux toward desired compounds, upregulates key biosynthetic enzymes, and alleviates pathway bottlenecks; hence, the yield of targeted secondary metabolites is significantly improved (Ramadani and Jadid, 2024). The shikimic acid, phenylpropanoid, and mevalonate pathways are major biosynthetic routes that are frequently studied in research to improve phytochemical production through precursor feeding. In a soilless system, the addition of chemical precursors into nutrient solutions has been suggested to boost the synthesis of valuable bioactive compounds. Soilless systems facilitate precise regulation of precursor concentration, exposure duration, pH, and delivery frequency. Nevertheless, excessive precursor levels can induce metabolic imbalance or phytotoxicity. Consequently, the type, dose, timing, and route of precursor delivery must be optimized for each medicinal plant and targeted metabolite. Several previous studies have reported that precursor feeding in a soilless system can significantly increase targeted metabolite levels. For example, exogenous supplementation of L-proline and L-ornithine, precursors involved in stachydrine hydrochloride biosynthesis, into the nutrient solution utilized for the cultivation of *Leonurus japonicus*. Houtt. In the floating technique, administering 0.5 mL^-1^ of L-proline for 96 hours before harvest increased the stachydrine hydrochloride concentration by over 50%, demonstrating the potential of precursor feeding to enhance specialized metabolite production in soilless cultivation systems (Song et al., 2012). Selecting inexpensive, soluble, and readily absorbable precursors is essential for the effective implementation of precursor feeding in soilless systems. When introduced into the nutrient solution, these precursors reach the roots directly, facilitating their uptake and subsequent metabolic conversion. Additionally, liquid cultivation enables repeated precursor supplementation and subsequent harvesting without the need for complete plant removal, potentially enhancing production efficiency. In hydroponic cultivation, phytochemicals are frequently released from the roots into the nutrient medium. Recovering secreted bioactive compounds from hydroponic nutrient media could provide a viable alternative to conventional plant harvesting. Paponov et al. (2021) reported that applying 10 µM MeJA to *H. perforatum* increased hypericin levels in root exudates by 88% compared with the control group. Moreover, data suggest that these two strategies can be utilized concurrently. *Datura innoxia* cultivated in a hydroponic solution with the precursors phenylalanine and ornithine exhibited a substantial enhancement in hyoscyamine and scopolamine levels in root exudates following root permeabilization with Tween 20 (Gontier et al., 2002). The permeabilization of roots may enhance the influx of secondary metabolite precursors into root cells, thereby enabling metabolite production and subsequent exudation into the liquid medium. These metabolites can subsequently be isolated from the medium for application in the manufacturing of medicines. Three saponin precursors: sodium acetate, geraniol, and D-mevalonolactone supplementation in hydroponically grown *Gynostemma* pentaphyllum boost the saponin (ginsenosides) content (Hu et al., 2025). Combining precursor supplementation with induced root permeabilization may facilitate the recovery of greater quantities of medicinally important compounds while eliminating the need for destructive plant harvesting (Atherton and Li, 2023). Overall, integrating precursor feeding with controlled soilless cultivation and metabolite recovery from the nutrient medium represents a promising approach for sustainable, precise, and non-destructive production of high-value phytochemicals. **Figure 7** shows a schematic illustration of how precursor feeding enhances phytochemical production in soilless farming.

**Figure 7:**
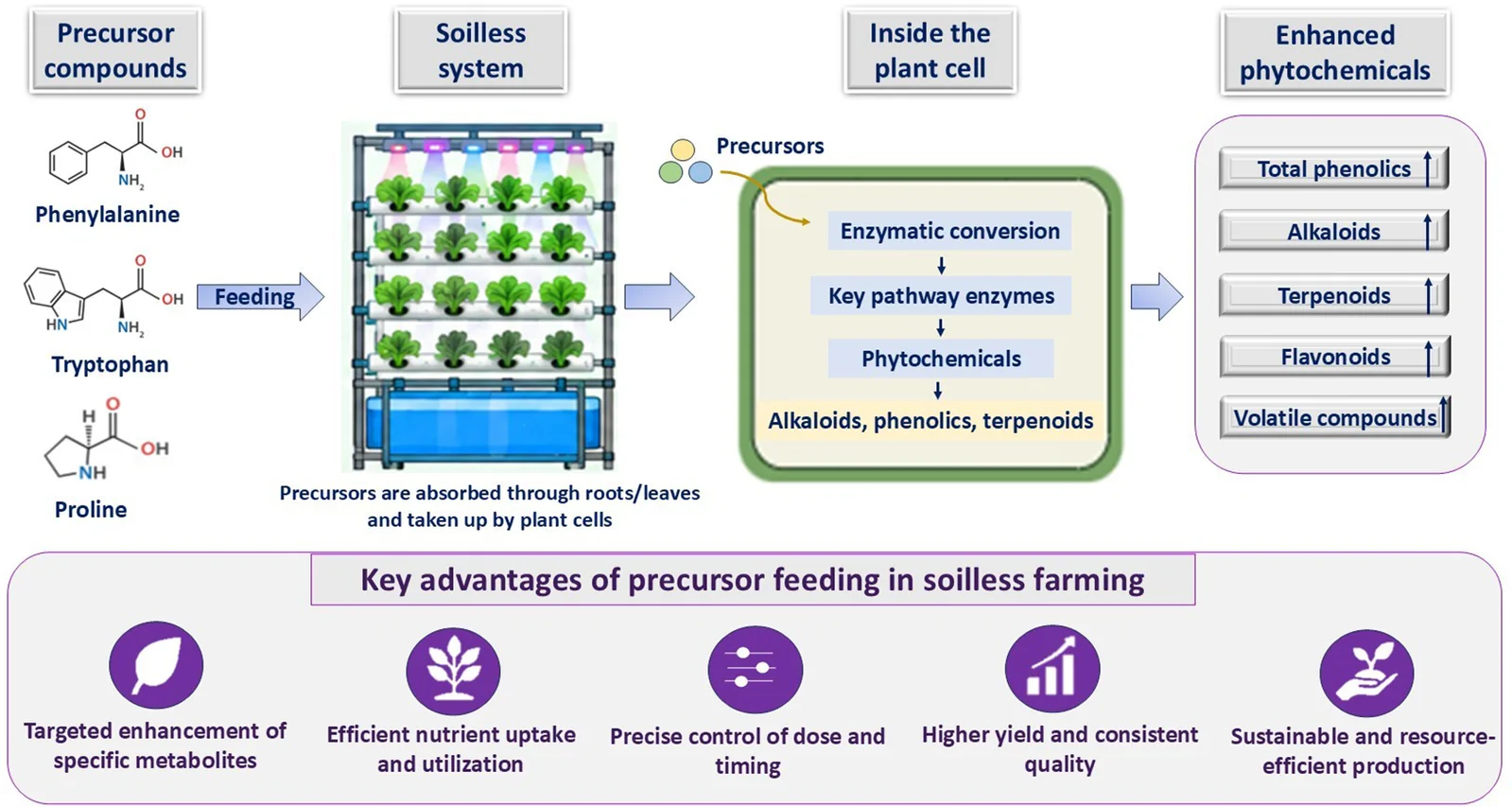
Schematic representation of the precursor feeding approach to boost phytochemical production in soilless farming systems.

## Efficient plant architecture by CRISPR-Cas technology

Vertical farming seeks to enhance productivity in restricted spaces by layering crops vertically, thereby maximizing yield per unit area (Delden et al., 2021). This approach is particularly suitable for leafy vegetables because of their compact architecture, rapid growth, and harvestable biomass; in contrast, fruit crops generally require greater vertical space and longer production cycles (Kwon, 2023; van Delden et al., 2021). Therefore, optimizing plant architecture is increasingly regarded as a key strategy to enhance the compatibility of fruit and other high-value crops with vertical farming systems. Genome editing offers a promising method for developing compact plant ideotypes through precise modification of genes that regulate plant height, stem elongation, branching, flowering, and canopy structure (Lim et al., 2025; Yu et al., 2025). Genome editing techniques, such as CRISPR/Cas (clustered regularly interspaced short palindromic repeats/CRISPR-associated protein), have emerged as viable methods for rapid, targeted trait enhancement (Mali et al., 2023; Verma et al., 2023). Multiplex CRISPR techniques further enable concurrent editing of the coding regions of multiple genes, as well as targeting of untranslated regions (UTRs) and cis-regulatory regions for precise transcriptional regulation (Byeon et al., 2021; Gupta and Beaudoin, 2021). In this context, genes involved in gibberellin biosynthesis and signaling represent important targets because gibberellins strongly regulate stem elongation and plant stature. In plants, *gibberellin 3-oxidase* (*GA3ox*) genes activate the gibberellic acid pathway and modulate stem length (Reinecke et al., 2013). Lim et al. (2025) employed multiplexed CRISPR gene editing to target the coding regions of three *SlGA3ox* genes (designated *SlGA3ox3*, *SlGA3ox4*, and *SlGA3ox5*). The *slga3ox4* single mutants had a marginal decrease in primary shoot length, resulting in a reduced height. The *slga3ox3 slga3ox4* double mutants exhibited a more compact shoot architecture with minimal physiological variation, suggesting they may be well-suited for vertical farming systems and offering prospective advantages for space-efficient agriculture. Furthermore, recent studies have shown that genome editing directly enhances production efficiency in commercial vertical farming systems. In another study, knocking out the tomato *SlGA20ox1* gene using CRISPR/Cas9 yields a favorable, vertical-farming-compatible plant structure characterized by reduced stem length and a dense canopy. The combination of *SlGA20ox1* with anti-florigen genes *SELF-PRUNING* (*SP*) and *SELF-PRUNING 5G* (*SP5G*) resulted in an increased compacted plant structure, expedited blooming, and coordinated fruit maturation. In commercial vertical farms, the *sp sp5g slga20ox1* triple mutant reduced spatial occupancy by 85%, shortened the harvest cycle by 16%, and increased effective output by 180%, thereby markedly improving productivity (Yu et al., 2025). These investigations provided experimental evidence that genome-edited architecture can influence not only plant morphology but also planting density, lighting requirements, harvest durations, and production efficiency.

Beyond plant height, recent research has extended CRISPR-based architectural engineering to canopy orientations. Fernandes et al. (2025) demonstrated that knockout of the *TAC1*-like gene (*Solyc10g009320*) using CRISPR/Cas9 generated markedly erect leaves, leaflets, and lateral shoots. The edited plants showed significantly reduced leaf insertion angles compared with wild type, confirming the role of *TAC1* in determining aboveground architecture. An erect canopy may enhance light distribution and space utilization in dense cultivation systems, underscoring the importance of modifying leaf and branch orientation and plant height. Recent editing of strigolactone-related genes *SlD14* and *SlMAX1* in tomato reduced plant height and promoted shoot formation. However, complete loss-of-function mutations caused excessive branching, source-sink imbalance, delayed ripening, and reduced fruit yield. A weak *sld14* allele led to a milder change in plant structure but left total fruit yield and harvest index unchanged. This suggests that using partial or allele-specific changes may be better for improving plant architecture than fully disrupting the gene (Lee et al., 2026). Kwon et al. (2020) propose that CRISPR-Cas9 editing of three specific genes, *SP5G*, which regulates flowering time; *SP*, which governs growth cessation; and *SlER*, which influences stem length, can convert any tomato genotype into a compact, early-yielding variety suitable for urban farming. This approach could transform the farming of diverse crops, such as fruits, medicinal and cosmetic herbs, and speciality high-value crops, profoundly influencing the advancement of modern farming (O’Sullivan et al., 2019). Collectively, these studies indicate a transition from traditional breeding for plant stature to precision engineering of plant architecture tailored to specific cultivation systems. Targeted editing of genes involved in gibberellin metabolism (*GA3ox*, *GA20ox*), flowering and growth cessation (*SP*, *SP5G*), branching and strigolactone signaling (*D14*, *MAX1*), and leaf orientation (*TAC1*) enables the development of compact, upright, and spatially efficient plant ideotypes. These architectural modifications can increase planting density, enhance canopy light interception, support multilayer stacking, and decrease the space, lighting, and labor demands of controlled-environment agriculture. However, the outcomes of architectural gene editing depend on the specific genes and alleles involved, and excessive reductions in stature or branching may adversely affect biomass allocation, reproductive development, and yield. Consequently, future genome-editing approaches should prioritize the optimization of plant architecture for specific cultivation platforms rather than focusing solely on maximizing compactness. Furthermore, integrating genome editing with phenomics and multi-omics approaches may support the development of compact medicinal-plant ideotypes optimized for biomass yield, resource-use efficiency, and specialized metabolite production. However, validation specific to crop and genotype remains necessary.

## Integrated and pathway-specific regulation of phytochemical production in soilless farming

The cultivation strategies described above work together as interconnected factors influencing plant growth, development, and metabolism. In soilless systems, carefully adjusting nutrients, light, water, temperature, elicitors, beneficial microbes, and precursor supply shapes the plant’s physiological and metabolic state. Plants sense these inputs through mechanisms that respond to nutrients, light, and stress. These responses converge in signaling networks involving reactive oxygen species (ROS), Ca²^+^, MAPK activations and phytohormone signaling. The resulting signals then control transcription factors, specific genes, enzyme activities, and precursor availability, which together modulate metabolic flux and phytochemical production (Zhao et al., 2005; Kumar et al., 2025b). This integrated perspective enables a shift from general enhancement of secondary metabolites to targeted, pathway-specific metabolic steering. For instance, manipulation of nutrient and light conditions can regulate carbon allocation and precursor availability, while elicitors and PGPMs can activate hormone- and defense-associated signaling pathways linked to specialized metabolism. By combining cultivation methods with gene expression analysis, enzyme assays, metabolite profiling, and metabolomics, researchers can identify regulatory points that lead to higher phytochemical production. Instead of optimizing each cultivation factor in isolation, using factorial or response-surface experimental designs helps reveal how nutrients, light, elicitors, and other inputs interact. By combining physiological, biochemical, transcriptomic, and metabolomic data, it becomes possible to identify pathway bottlenecks and distinguish between higher metabolite concentrations and greater overall metabolite productivity. Furthermore, IoT-based monitoring and ML can integrate environmental and plant-response data to predict optimal growing conditions (Sudha and Loret, 2026; Sharma et al., 2026). These tools can also adjust nutrients, light, or other factors as plants grow and their needs change. Thus, in the future, soilless phytochemical production should move away from focusing on single factors and instead use integrated, pathway-based metabolic control. This approach offers a more systematic way to develop reliable, targeted phytochemical production systems.

## Conclusion and prospects

Soilless farming can significantly contribute to the long-term viability of food and quality biomass production in densely populated areas. The need for clean, fresh food is increasing significantly due to population growth, and individuals will adopt innovative plant cultivation methods to meet this growing demand (Lakhiar et al., 2018). Soilless farming offers numerous benefits compared with conventional farming, particularly across sustainable, environmental, social, and economic dimensions (Fussy and Papenbrock, 2022). For example, a sustainable food-producing system that enables year-round crop production, much higher yields, and safeguards against droughts, floods, and pests. In residential and commercial buildings, implementing innovative temperature-control units and natural-light management systems has effectively reduced energy consumption and minimized greenhouse gas emissions, which are widely used in plant factories (Germer et al., 2011; Fussy and Papenbrock, 2022). These factors have enabled the establishment of local food production in densely populated urban areas, where the increased demand for food and herbs cannot be addressed (Thomaier et al., 2015). The review emphasizes the importance of soilless farming and examines the latest technological advancements in this field. There is a growing demand for multidisciplinary investigation and collaboration to encourage collective thinking among the several disciplines involved in the development of fully automated soilless farming systems.

Recent advances in dynamic LED lighting, spectral modulation, precision nutrient management, precursor feeding, microbial biostimulants, and nano-elicitation further expand the potential of soilless systems for targeted production of phytochemicals. Furthermore, CRISPR/Cas technology is a powerful tool with considerable potential to alter genomes more quickly, swiftly, and precisely, enhancing trait improvement and shoot architecture for maximum utilization of space in vertical farming (Kwon, 2023; Yu et al., 2025). Moreover, precursor feeding approaches combined with microbial biostimulants in soilless agriculture enhance plant growth and phytochemical synthesis (Mubeen et al., 2023; Hu et al., 2025). Considering the current situation, in which soilless farming is primarily used to cultivate leafy greens, herbs, lettuces, tomatoes, and peppers, it is imperative to develop innovative solutions that can increase productivity and profitability across a diverse range of crops (Erekath et al., 2024). Rigorous scientific investigations in medicinal plant cultivation demonstrate that the production and accumulation of secondary metabolites in plant roots and rhizomes can be achieved through soilless cultivation by adjusting mineral nutrition, biostimulant and precursor feeding strategies, and managing growing conditions (Rattan et al., 2022). The composition of the nutritional solution and environmental conditions both affect tissue quality and biomass production. Soilless farming is well-suited for cultivating plants that contain specialized metabolites, which can be utilized in the manufacture of pharmaceuticals, nutraceuticals, or skin care products (Thakur et al., 2019; Sharma et al., 2024). The soilless farming industries worldwide are expected to experience significant expansion in the present and near future. Developing cost-effective soilless farming systems is crucial (Singh and Hati, 2024). It was created to enhance commercial soilless farming systems and minimize initial setup and ongoing maintenance costs. Currently, soilless farming is being influenced by industry, with a significant number of independent startups (Fussy and Papenbrock, 2022). Academic organizations face insufficient funding for soilless farming research and the absence of standards in these high-tech systems and supply chains, caused by each firm developing its method, obstructing the optimum efficiency in these systems (Beacham et al., 2019). Furthermore, a significant portion of the data used to assess the viability of soilless farming, such as crop production data, is derived primarily from promotional materials or speculation rather than rigorous scientific research. Additionally, this data may not be accessible to the public. This circumstance requires further investigation into the feasibility of soilless farming for efficient food production. Soilless farming is a sector where the company would benefit from greater collaboration with academic institutions to fully exploit its potential and assess the likelihood of future growth as a sustainable means of food and biomass production. Increasing awareness through the establishment of nurseries and visitor centers, and by involving the local community, governmental organizations, and small-business owners, can significantly contribute to the expansion of vertical farms and hydroponic farming (Srinivasan and Yadav, 2024; Petrovics et al., 2024). Future research should therefore prioritize energy-efficient lighting, crop-specific dynamic lighting strategies, integrated light-nutrient-elicitor management, standardized cultivation protocols, techno-economic assessment, life-cycle evaluation, and scalable automation.
